# The role of cannabinoid agonists and antagonists on folliculogenesis and evolutionary events in the mouse ovary

**DOI:** 10.22038/ijbms.2025.85417.18468

**Published:** 2025

**Authors:** Vida Mirzaie, Touba Eslaminejad, Fatemeh Sheikhbahaei, Shayan Vafaei, Fatemeh Nabipour, Mina Behzadi, Seyed Noureddin Nematollahi-Mahani

**Affiliations:** 1 Department of Anatomical Science, Afzalipour School of Medicine, Kerman University of Medical Sciences, Kerman, Iran; 2 Department of Anatomy and Cell Biology, School of Medicine, Mashhad University of Medical Sciences, Mashhad, Iran; 3 Department of Pathology, School of Medicine, Kerman University of Medical Sciences, Kerman, Iran; 4 Kerman Neuroscience Research Center, Neuropharmacology Institute, Kerman University of Medical Sciences, Kerman, Iran; 5 Kerman Neuroscience Research Center (KNRC), Institute of Neuropharmacology, Kerman University of Medical Sciences, Kerman, Iran

**Keywords:** Cannabinoid, Cannabinoid agonist, Cannabinoid antagonist, CB1 receptor, CB2 receptor, Folliculogenesis, Ovary

## Abstract

**Objective(s):**

Cannabinoids, derivatives of *Cannabis sativa* L., can activate the endocannabinoid system via two endogenous receptors, CB1 and CB2. This system is crucial in regulating folliculogenesis, fertility, and reproductive function. This study investigated the potential effects of cannabinoid agonists and antagonists on ovarian health and function in female mice.

**Materials and Methods:**

80 NMRI mice were divided into 10 groups. Treatment groups received CB1 or CB2 agonists, antagonists, or their combinations for five days. The animals were then sacrificed, the ovaries were excised and weighed, and their volume was measured. Total RNA was extracted from the left ovary for qPCR analysis, while the right ovary was fixed in Bouin’s solution for histological evaluation following H&E staining.

**Results:**

Treatment with CB1/CB2 agonist+CB1 antagonist (W102+AM251) decreased the level of NAPE-PLD (a key factor in the production of endocannabinoids in cells) and increased the level of FAAH (responsible for cannabinoid degradation) genes compared to all groups. CB2 antagonist (AM630) increased the number of primary, preantral, and antral follicles, the volume and weight of ovaries, and estrogen levels. Meanwhile, the CB1 antagonist (AM251) significantly increased microvascular density in the ovaries.

**Conclusion:**

Cannabinoids modulate ovarian physiology and folliculogenesis, with CB2 receptors playing a particularly significant role. Antagonism at CB2 appeared to differentially affect cannabinoid-metabolizing enzymes in ovarian follicles and differentially affect their maturation. However, our preliminary novel findings in mice require human studies before clinical application.

## Introduction

Plant-derived compounds known as cannabinoids (phytocannabinoids) can be naturally produced in the body or synthesized chemically. Both endogenous cannabinoids (unsaturated fatty acid derivatives) and exogenous ligands activate the endocannabinoid system (ECS) by binding to cannabinoid receptors (1). Cannabinoids have been used for centuries for therapeutic and recreational purposes and regulate various physiological processes, including pain, neural development, reproduction, and hormone secretion (2). Cannabinoids derived from *Cannabis sativa L.*, such as marijuana and hashish, are classified as illegal drugs in many countries and have seen increased use among women in recent years (3). The ECS is modulated by enzymes such as N-acyl phosphatidylethanolamine-specific phospholipase D (NAPE-PLD) and fatty acid amide hydrolase (FAAH). NAPE-PLD catalyzes the hydrolysis of NAPE, initiating the primary and best-known pathway for endocannabinoid synthesis, while FAAH is responsible for cannabinoid degradation (4).

The ECS consists of at least two G protein-coupled receptors, CB1 and CB2, which have been identified in various species, including mice and humans (5, 6). CB1 receptors are primarily found in neural tissues, ovaries, endometrium, testes, vas deferens, and bladder, whereas CB2 receptors are predominantly expressed in immune cells, brain, bones, and the reproductive system, including the ovaries (7, 8). El-Talatini *et al*. reported that CB2 receptors are widely expressed in the ovarian medulla and cortex. In contrast, CB1 receptors are highly expressed in the granulosa cells of follicles at different stages and in the corpus luteum and corpus albicans (9). Cannabinoid receptors in specific brain regions, such as the hypothalamus, are linked to the hypothalamic-pituitary-ovary axis and the hormonal regulation of reproductive functions (10-12). ECS components have been detected in female reproductive fluids, ovaries, fallopian tubes, and the uterus, particularly in the endometrium (1, 13). The presence of cannabinoid receptors in the female reproductive system suggests that ECS dysregulation may lead to abnormal modulation of key reproductive processes (14-17). Studies indicate that cannabinoid receptor expression in reproductive tissues fluctuates throughout the menstrual cycle in response to steroid hormone levels (18, 19). Estrogen, a key steroid hormone with membrane and nuclear receptors, plays a crucial role in regulating female reproductive functions (20). Cannabinoids and endocannabinoids exhibit sex-specific effects on various biological functions, including emotion, cognition, receptor binding, and THC metabolism. These differences contribute to the varying sensitivities observed between sexes and among individuals. Additionally, estradiol (E2) has been shown to regulate the ECS, further demonstrating these sexually dimorphic effects (21). Given that reproductive organs, including the uterus, express ECS elements essential for reproductive physiology, and that estrogen plays a key role in this process, estradiol may directly modulate ECS activity in female reproductive tissues (22). The expression of cannabinoid receptors also appears to be influenced by estradiol levels. Research suggests that responses to exogenous cannabinoids vary with steroid hormone fluctuations (23, 24). Cannabinoids have been shown to decrease plasma estrogen levels in both males and females by affecting serum luteinizing hormone (LH) and gonadotropin-releasing hormone (GnRH) at the hypothalamic-pituitary axis (25, 26). Several signaling molecules, including adenylate cyclase (AC), protein kinase A (PKA), epidermal growth factor (EGF), and cAMP response element-binding protein (CREB), are influenced by both cannabinoids and estrogen, explaining their opposing effects (27). 

Overall, these findings indicate that cannabinoids may influence reproductive events, fertility, and endocrine regulation in females (14, 15). They can disrupt the menstrual and follicular cycles and affect sex hormone secretion (28). Some studies even suggest that cannabinoid exposure may contribute to infertility (15, 29). However, limited research exists on the impact of cannabinoid receptor agonists and antagonists on folliculogenesis, follicular maturation, and growth. Since ovarian hormones significantly influence the sex-specific effects of exogenous cannabinoids (30, 31), we have designed a comprehensive study to evaluate folliculogenesis and oocyte development following cannabinoid receptor stimulation and inhibition. This study will employ various cannabinoid agonists and antagonists while measuring serum estradiol levels. The findings may provide valuable insights into the complex mechanisms of folliculogenesis and oocyte maturation, potentially aiding in the treatment of ovarian disorders and infertility.

## Materials and Methods

The methods were approved by the Kerman University of Medical Sciences ethics committee (approval code: KMU-910428), and the animals were treated in compliance with the rules of the same committee. Materials were purchased from Sigma Company (Sigma-Aldrich, MO, USA) unless stated otherwise. WIN 55, 212-2 mesylate (A11932-50), AM251 (A12914-50), and AM630 (A14993) were purchased from AdooQ® Bioscience (USA).

### Animals

Eighty NMRI female mice (5–9 weeks old, 20–30 g) and five male mice were purchased from the animal house of Afzalipour School of Medicine, Kerman, Iran. Animals had free access to water and rodent food (Razi Institute, Tehran, Iran) and were maintained on a 12-hr dark/light cycle. To minimize the effect of confounding factors in the research and equalize the level of basal hormones, we synchronized the estrus cycle of the animals as shown below. Five female mice and one male mouse were placed in a special self-designed two-part cage so that the female mice would not be in direct contact with male mice for one week. Then, a vaginal smear was prepared and the animals in the proestrus stage (presence of small, round, nucleated epithelial cells, no neutrophils, low numbers of large epithelial cells, and keratinized anucleated cells (32) were selected and randomly divided into different groups with eight mice in each group. Animals received drugs intraperitoneally from day one for 5 days to cover a complete estrous cycle in mice and to ensure treatment assessment at all hormonal phases, according to the grouping and dose details (Table 1). This approach minimizes variability due to hormonal fluctuations and aligns with established methodologies in reproductive research in mice (33).

### Experimental design

 A total of ten groups of female mice, each group consisting of eight mice, were used. The injection volume, dose of active components, and corresponding excipients are summarized in Table 1. Mice were exposed to different CB1 and CB2 agonists, antagonists, and their combination in various groups to assess the drug-induced effects. To dilute the drugs, calculations for W102(10 mg/kg) was performed as follows;

Total number of mice that should be given W102 in all groups: 24 weight of a sample mouse: 30 g

Duration of treatment: 5 days, therefore 40 mg of W102 compound is needed for 4 kg weight of mice.

The volume injected into the sample mouse: 300 µl

Total volume for 24 mice and 5 times treatment: 36 ml

Each vial of W102 (25 mg) was dissolved in 1.8 ml of DMSO (concentration 1%), and then its volume was increased to 18 ml with PBS (two vials were used in total). Therefore, each 1 µl of solution contains 1 µg of W102. Thus, each mouse receives 300 µl of the diluted solution daily (300 µg). Other compounds were calculated based on their dosage in the same way. The vehicle group received 300 µl 1% DMSO to nullify the probable effects of DMSO. At the end of the treatment periods, the animals were sacrificed by cervical dislocation, blood samples were taken from the left ventricle, ovaries were removed, and the following experiments were performed. 

### Group characteristics

As mentioned in Table 1, the different treatments used in this study have different effects on cannabinoid receptors. The characteristics of each group are summarized below for better follow-up.

Group 1: activated CB1/CB2 receptors by W102

Group 2: deactivated CB1 receptor by AM251

Group 3: deactivated CB2 receptor by AM630

Group 4: activated CB1 receptor by A9719

Group 5: activated CB2 receptor by G1421

Group 6: activated CB1/CB2 receptors by W102 and decactivated CB1 receptor by AM251

Group 7: activated CB1/CB2 receptors by W102 and decactivated CB2 receptor by AM630 

Group 8: activated CB2 receptor by G1421 and deactivated CB1 receptor by AM251

Group 9: activated CB1 receptor by A9719 and deactivated CB2 receptor by AM630

Group 10: DMSO control 

### Specimen collection

After five days of treatment, animals were sacrificed by cervical dislocation. Blood was collected for serological analysis into clean tubes, and the ovaries were isolated after abdominal wall incision. Ovary weight was recorded by a sensitive digital scale (A&D, GF-300, Japan), and different dimensions of the ovaries were measured using a digital caliper (Mitushi PRO-CAL). Ovarian volume was calculated using the prolate ellipsoid formula (L × H × W × 0.523; where L was length, H was height and W was weight). The right ovary was placed in Bouin’s fixative solution for histological examinations, and the left ovary was frozen (-70˚c) for RNA extraction and gene expression analysis.

### Gene expression studies

***RNA extraction***

Total RNA was isolated from each ovary as a reference for the genes evaluated by adding 1 ml of RiboEx™ reagent according to the manufacturer’s instructions. A A_260_:A_280 _ratio of 1.81 ± 0.06 was obtained after evaluation by a UV spectrophotometer (Thermo Scientific™ NanoDrop 2000). cDNA was synthesized using a cDNA synthesis kit (Parstous, Iran), according to the manufacturer’s instructions. 0.1 µg of total RNA was used for every first-strand cDNA synthesis: primer annealing at 25 °C for 10 min, denaturation at 47 °C for 60 min, and heat inactivation at 85 °C for 5 min. 

*Real-time PCR and gene expression*

Real-time PCR was performed using Sina SYBER blue reaction mix without low ROX (Sina Clone) in a magnetic induction cycler (BMS, Australia) Real-time PCR system, using 1.5 μl of each cDNA sample, and 10 pmol of each primer. The reaction program was 95 °C for 7 min, followed by 40 cycles of 95 °C for 15 sec, 65 °C for 20 sec, and 72 °C for 35 sec. The C_T_ (threshold cycle) values were calculated using the 2^-∆∆CT^ method (40). *β-actin* and *GAPDH* were used as the endogenous reference genes for normalizing the fold change in gene expression. The intrinsic expression of the DMSO group was normalized by the reference genes and set to one. *FAAH* and *NAPE-PLD* expression levels were quantified. The primer sequences were designed with Primer3Plus software and are summarized in Table 2.

### Histological studies

After tissue processing of Bouin’s-fixed ovaries, dehydration, clearing, and paraffin embedding, each ovary was serially sectioned (5 µm thick) using a rotary microtome (41). Eight sections were selected from each ovary (every 250 µm) and stained with hematoxylin-eosin. The sections were evaluated under a light microscope (Olympus IX51, Tokyo, Japan) to determine the number of luteal bodies, micro-vessels, and follicles containing oocytes with a germinal vesicle (to avoid double-counting of each follicle). Follicles were classified as follows: 1. primary follicle: an oocyte surrounded by one layer of cuboidal granulosa cells, 2. secondary follicle: an oocyte surrounded by two or three layers of cuboidal granulosa cells without any antral space, 3. Preantral follicle: an oocyte surrounded by more than four layers of granulosa cells with one or more independent antral spaces, and 4. antral follicle: an oocyte surrounded by multiple layers of cuboidal granulosa cells with a defined large antrum (42). 

### Estradiol measurement

After 5 days of treatment, the animals were sacrificed by cervical dislocation. Blood was quickly aspirated from the left ventricle, centrifuged at 2000–2500 g for 10 min; the serum was collected and kept at -20 °C for subsequent experiments. Estradiol was measured using appropriate laboratory DRG Estradiol kits for mice (LIAISON Estradiol (310400) kit (Diasorin Inc, USA)) as recommended by the manufacturer. The limit of detection (LOD) level was found to be 12 pg/ml. Inter- and intra-assay coefficients of variation were below 5 and 9.3%, respectively.

### Statistical analysis

All experiments were performed in triplicate. Data were analyzed by ∆∆ Ct method and expressed as mean ± SEM. Quantitative variables were normally distributed and assessed using one-way ANOVA with Tukey’s post hoc test. Microsoft Excel 2019 and GraphPad Prism software were used to manage data and draw graphs. Differences were considered statistically significant at a 0.05 threshold.

## Results

*Gene expression*

**Figure 1:**
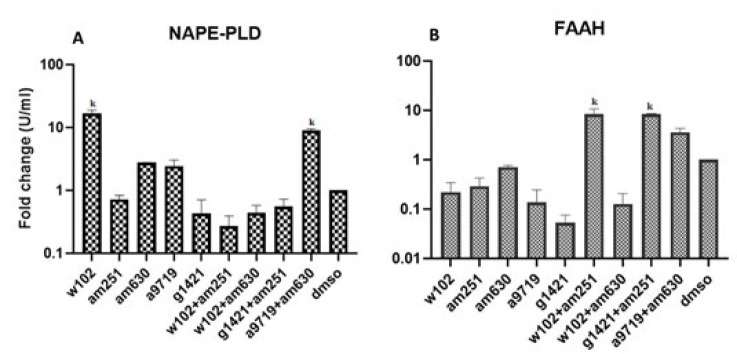
Ovarian expression of NAPE-PLD and FAAH genes among different groups in NMRI mice after cannabinoid agonist and antagonist administration

Gene expression analysis in ovaries of different groups showed that the expression of NAPE-PLD in the groups treated with W102 and A9719+AM630 was significantly (*P*<0.05) higher than in other groups. The lowest expression of NAPE-PLD was in the group treated with W102+AM251. Analysis of FAAH gene expression indicated that groups treated with W102+AM251 and G1421+AM251 had significantly (*P*<0.05) higher expression levels than other groups. In contrast, the lowest expression of FAAH was observed in the group treated with G1421 (Figure 1, supplementary 1).

*Histological findings*

**Figure 2:**
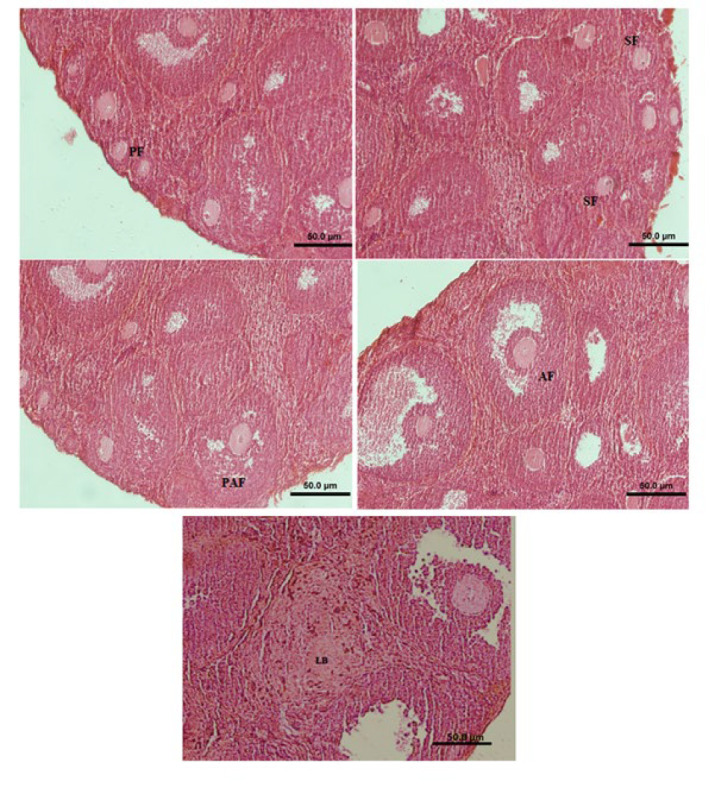
Primary follicle (PF), Secondary follicle (SF), Preantral follicle (PAF), Antral follicle (AF) and Luteal body (LB)

**Figure 3:**
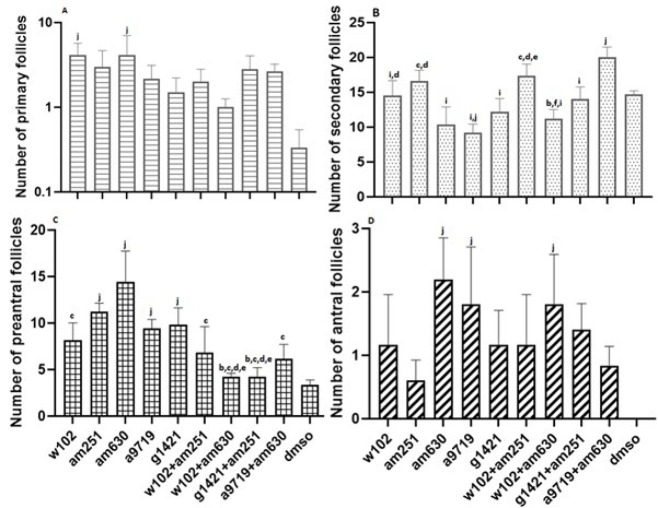
Quantification of (A) primary, (B) secondary,(C) preantral, and (D) antral follicles across different experimental groups of NMRI mice after cannabinoid agonist and antagonist administration

The number of different follicles in the ovaries (shown in the histological panel) of all groups was counted (Figure 2). The histological panel of all types of follicles is presented in the manuscript. The number of primary follicles increased in all groups compared with the DMSO group, and a significant (*P*<0.05) difference was observed in the groups treated with W102 and AM630 (Figure 3, supplementary 2). 

The number of secondary follicles in the animals treated with A9719+AM630 was significantly (*P*<0.05) higher than the number of secondary follicles in the animals treated with W102, AM630, A9719, G1421, W102+AM630, G1421+AM251 and DMSO, while the animals treated with A9719 had significantly (*P*<0.05) fewer number of secondary follicles than the animals treated with W102, AM251, W102+AM251 and DMSO. The number of secondary follicles in the animals treated with AM630 was also significantly (*P*<0.05) lower than that of the animals treated with AM251 and W102+AM251. Also, a significant difference (*P*<0.05) was observed between the animals treated with AM251 compared with the animals treated with W102+AM630, G1421, W102+AM251, W102+AM251, and W102+AM630 (Figure 3, supplementary 2). 

The number of preantral follicles in animals treated with AM630 was significantly (*P*<0.05) higher than in all groups except those treated with AM251, A9719, and G1421. The number of preantral follicles was also significantly (*P*<0.05) higher in animals treated with AM251, A9719, and G1421 than in animals treated with W102+AM630, G1421+AM251, and DMSO (Figure 3, supplementary 2). 

There were no antral follicles in the ovaries of DMSO-treated mice. In contrast, the number of antral follicles in animals treated with AM630, A9719, and W102+AM630 was significantly (*P*<0.05) higher than that of the DMSO group (Figure 3, supplementary 2).

**Figure 4:**
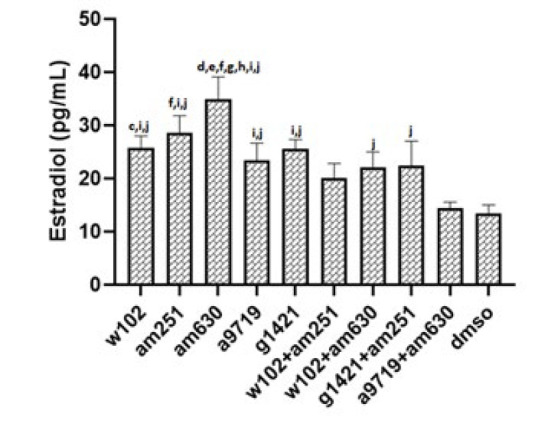
Number of (A) luteal bodies, (B) microvessel counts, and ovarian (C) volume and (D) weight across different experimental groups of NMRI mice after cannabinoid agonist and antagonist administration

In general, the number of luteal bodies increased in all groups compared to the DMSO group. The number of luteal bodies in animals treated with W102 was significantly (*P*<0.05) lower than the number of luteal bodies in G1421-treated animals and W102+AM251-treated animals. G1421-treated animals had a significantly higher number of luteal bodies than the W102+AM630, G1421+AM251, and DMSO-treated animals. The number of luteal bodies was also significantly (*P*<0.05) higher in animals treated with W102+AM251 and A9719+AM630 compared to the DMSO-treated group (Figure 4, supplementary 2). 

The number of micro-vessels in AM251-treated animals was significantly (*P*<0.05) higher than that of all groups. The values in the G1421-treated animals were also significantly (*P*<0.05) higher than those in A9719 and DMSO-treated animals (Figure 4 and Supplementary 3). 

In animals treated with AM630, ovarian volume was significantly (*P*<0.05) higher than in the A9719, W102+AM251, and W102+AM630-treated animals. Also, it was significantly (*P*<0.05) higher in animals treated with A9719+AM630 in comparison to the ovary volume in the W102+AM630 and A9719-treated animals (Figure 4 and supplementary 3). 

Comparing the weight of ovaries in different groups indicated that all groups had significantly (*P*<0.05) less weight than the DMSO-treated group. However, animals treated with W102 and AM630 had significantly (*P*<0.05) higher ovary weight than those treated with A9719, W102+AM251, W102+AM630, and G1421+AM251. In contrast, animals treated with A9719, W102+AM251, and W102+AM630 had significantly (*P*<0.05) lower ovary weight than animals treated with A9719+AM630 (Figure 4 and supplementary 3).

*Estradiol measurement*

**Figure 5:**
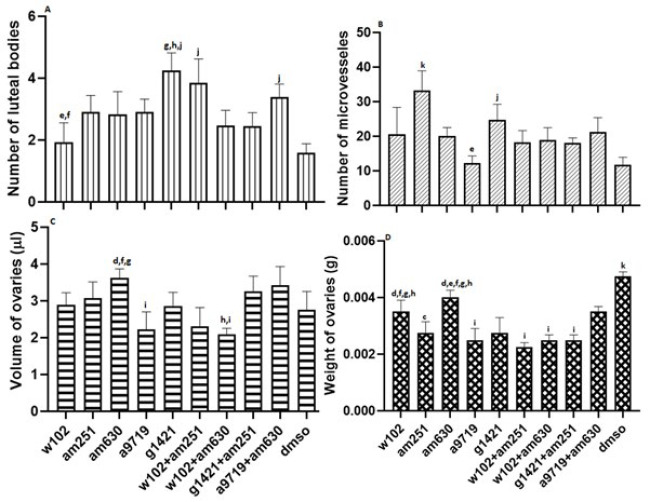
Estradiol hormone levels across different experimental groups of NMRI mice after cannabinoid agonist and antagonist administration

After evaluating the estradiol level, the mice with the same range (estradiol level of metestrus to estrus stage) were included in the statistics according to the normal estradiol level in mice (43). According to this classification, only one mouse in W102, one in W102+AM630, and two in A9719+AM630 groups were excluded as their estradiol level was out of range. Therefore, more than 90 percent of animals were at the same estrus cycle stage. Serological analyses indicated that estradiol levels significantly (*P*<0.05) increased in all groups except in animals treated with A9719+AM630. The estradiol level in AM630-treated animals was significantly (*P*<0.05) higher than all groups except the AM251-treated group. Treatment of animals with A9719+AM630 resulted in a significantly (*P*<0.05) lower level of estradiol compared with groups treated with W102, A9719, and AM251 (*P*<0.05) (Figure 5 and supplementary 3).

## Discussion

Cannabinoid receptors CB1 and CB2 as members of GPCRs** (**G protein-coupled receptors) (5, 44) are found in different organs, including the Ovaries (1). IHC** (**Immunohistochemical)staining has also revealed that NAPE-PLD, FAAH, and CB receptors are localised in human ovarian follicles (9, 45). Cannabinoids can exert different effects on each person by triggering the activity of different receptors and involving different pathways. This fact is supported by reports showing the different effects of cannabinoids on different individuals (46-50). Our findings, summarized in Table 3, suggest that CB1 receptor agonists (A9719) administration and CB2 receptor antagonists (AM630) alone or in combination lead to the highest levels of *NAPE-PLD* expression, indicating an increase in endocannabinoid synthesis. Conversely, FAAH expression was significantly elevated in groups where the CB1 receptor was inhibited while the CB2 receptor was activated, suggesting a compensatory mechanism for cannabinoid degradation. Cannabinoid production in the body is related to CB1 receptor activation and CB2 receptor deactivation. Our results showed that activation of each receptor alone decreases *FAAH* level (A9719 and G1421). In contrast, deactivation of the CB1 receptor in combined groups, where the CB2 receptor had been activated (W102+AM251 and G1421+AM251), increases *FAAH* level, suggesting that *FAAH* needs a deactivator that acts as a trigger to increase its activity and hydrolyze excess cannabinoids in the body. Interestingly, in ovarian follicles, the CB2 receptor is expressed more than the CB1 receptor (note that both receptors are present in ovarian follicles) (9, 51). This conclusion is confirmed by the results of our study, where the most significant changes in the various studied parameters were observed when the animals received CB2 receptor antagonists (as a general conclusion). 

For ease of survey and a better understanding of histological data, the period of follicular maturation was divided into two episodes of follicular growth, including primary/secondary follicle formation and antral follicle development, including preantral/antral follicles. In this study, we focused on the effect of cannabinoid agonists and antagonists on growing follicles; therefore, the ovarian reserve (primordial follicles) was not estimated. The data indicate distinct CB1 and CB2 receptor modulation effects on folliculogenesis. Lim *et al*. demonstrated that exposure of female mice to cannabinoids (THC) led to improper activation of the ovarian endocannabinoid system and negatively affected ovarian reserve by reducing primordial and primary follicles. In addition, they found that exposure of female mice to cannabinoids led to DNA damage in primary follicles (52). Since in our study, W102 administration increased the number of primary follicles, we may hypothesize that these differences may be due to differences in the effects of the receptor agonists versus the partial agonist THC or to the use of adult mice in our study, as opposed to adolescents in the cited paper. Our data revealed that blocking CB2 receptors (AM630 group) enhanced follicular maturation, evidenced by increased preantral and antral follicles. We observed a complete absence of antral follicles in the DMSO group, although DMSO was only used in a solvent dose. This finding is consistent with previous studies indicating that DMSO can adversely affect follicular development. For example, Li *et al*. reported that 3% DMSO treatment inhibited cumulus expansion and increased nuclear abnormalities in porcine oocytes (53). Additionally, Cecconi *et al*. found that approximately 50% of DMSO-treated follicles showed signs of degeneration after one day of culture (54). 

 Pirone *et al*. reported that CB1 was not expressed in early follicles of cats’ ovaries but was detected in antral follicles’ germinal cells. Thus, modulation of CB1 could regulate fertility (55). Our findings on the CB1 receptor showed that the least number of antral follicles was detected after CB1 deactivation (AM251 group). On the other hand, CB1 receptor activation (A9719 group) significantly reduced secondary follicle counts, suggesting that CB1 signaling may predominantly regulate later stages of follicular development. These findings align with previous studies indicating that CB1 receptors are more expressed in granulosa cells of mature follicles, while CB2 receptors are widely distributed throughout ovarian tissue. A reduction in the number of microvessels following treatment of animals with CB1 receptor agonist (A9719 group) may suggest a role for CB1 in ovarian angiogenesis. However, this effect can be compensated by deactivating the CB2 receptor (the highest number of antral follicles in the AM630 group, Table 3). 

The various effects of cannabinoids on the female reproductive system have made this field of study very ambiguous. Gammon *et al*. demonstrated that cannabinoids (CB agonists) negatively affect the hypothalamic-pituitary-ovary axis and reduce steroid hormones as well as gonadotropin-releasing hormones (10). Examining the level of estradiol in the present study has also shown that, notably, estradiol levels increased following CB2 receptor antagonism(AM630), indicating a negative regulatory role of CB2 in ovarian steroidogenesis. This is consistent with prior research showing that cannabinoids suppress gonadotropin-releasing hormone (GnRH) and estrogen production, thereby disrupting endocrine homeostasis. In our study, the weight and volume of the ovaries were significantly higher in the CB2 antagonist-treated group (AM630), reinforcing the notion that CB2 inhibition promotes follicular development. Previous reports have also suggested that CB2 receptors exert suppressive effects on ovarian growth, which may explain the observed increase in ovarian size upon CB2 blockade (56). Similar results have been reported after administration of cannabinoids to a mouse model of OHSS (Ovarian Hyper Stimulation Syndrome) (57). 

Our findings indicate that CB2 receptor antagonism enhances follicular growth, while CB1 receptor activation impairs follicular progression. These results highlight the complex regulatory role of cannabinoid signaling in ovarian function and suggest that CB2 receptor inhibition may have therapeutic potential for ovarian disorders. However, further investigations, including dose-response studies and clinical trials, are needed to confirm the translational relevance of these findings to human reproductive health.

**Table 1:** Study groups and dosage details of cannabinoid agonist and antagonists administered to NMRI mice

	**Group**	**Administered doses**	**Molar doses**	**Drug solvent**	**Drug characteristics**
1	WIN 55,212-2 mesylate, A11932-50, Adooq, 50MG (W102) (34) MV: 500.64 g/mol	10 mg/kg	20 µmol	12 mg/ml (DMSO)	CB1/CB2 agonist
2	AM251, A12914-50, Adooq, 50MG (A6226) (35) MV: 554.49 g/mol	10 mg/kg	18 µmol	<10 mg/ml (DMSO)	CB1 antagonist
3	AM630, 10mg, A14993, Adooq, 10mg (SML0327) (36, 37) MV: 502.99 g/mol	5 mg/kg	9.94 µmol	<5 mg/ml (DMSO)	CB2 antagonist
4	ACEA, A9719, 25MG, Sigma (ACEA) (38) MV: 389.51 g/mol	5 mg/kg	12.83 µmol	<10 mg/ml (DMSO)	CB1 agonist
5	GW405833 hydrochloride, G1421, 25MG, Sigma (G1421) (39) MV: 389.87 g/mol	10 mg/kg	25.64 μmol	<10 mg/ml (DMSO)	CB2 agonist
6	W102 + AM 251	10 mg/kg + 10 mg/kg			
7	W102 + AM630	10 mg/kg + 5 mg/kg			
8	GW405833 + AM251	10 mg/kg + 10 mg/kg			
9	A9719 + AM 630	5 mg/kg + 5 mg/kg			
10	Vehicle	300 µl DMSO 1%			

**Table 2:** Forward and reverse primers used in the study to evaluate FAAH, NAPE-PLD, GAPDH and B-actin gene expression in ovaries of NMRI mice after cannabinoid agonist and antagonist administration

**Gene **	**Forward (5** **ꞌ** **→** **3** **ꞌ** **)**	**Reverse (5** **ꞌ** **→** **3** **ꞌ** **)**
***FAAH***	CAGGAGATCATGGTGCTGAG	CAAGGCCTCTGAATCCAGGT
***NAPE-PLD***	GTGAGCCTCCCTGCTGTTAG	CTGAATTCTGGCGCTTTCTC
***GAPDH***	CGTCCCGTAGACAAAATGGT	GAATTTGCCGTGAGTGGAGT
***β-actin***	CAGCTTCTTTGCAGCTCCTT	CACGATGGAGGGGAATACAG

(k) significant difference from all groups, (*P*<0.05)

Statistical significance (*P*<0.05) is indicated as follows:(b) significantly different compared with AM251 group;(c) significantly different compared with AM630 group;(d) significantly different compared with A9719 group;(e) significantly different compared with G1421 group;(f) significantly different compared with W102 + AM251 group;(g) significantly different compared with W102 + AM630 group;(i) significantly different compared with A9719 + AM630 group;(j) significantly different compared with DMSO group.

Statistical significance (*P*<0.05) is indicated as follows:(c) significantly different compared with the AM630 group;(d) significantly different compared with the A9719 group;(e) significantly different compared with the G1421 group;(f) significantly different compared with the W102 + AM251 group;(g) significantly different compared with the W102 + AM630 group;(h) significantly different compared with the G1421 + AM251 group;(i) significantly different compared with the A9719 + AM630 group;(j) significantly different compared with the DMSO group;(k) significantly different compared with all groups.

Statistical significance (*P*<0.05) is indicated as follows:(c) significantly different compared with the AM630 group;(d) significantly different compared with the A9719 group;(e) significantly different compared with the G1421 group;(f) significantly different compared with the W102 + AM251 group;(g) significantly different compared with the W102 + AM630 group;(h) significantly different compared with the G1421 + AM251 group;(i) significantly different compared with the A9719 + AM630 group;(j) significantly different compared with the DMSO group.

**Table 3:** Overview of the effects of administration of cannabinoid agonist and antagonists on different ovarian parameters of NMRI mice

**Groups/ parameter studied and influenced **	**Activation of CB1R/CB2R**	**deactivation of CB1R**	**deactivation of CB2R**	**activation of CB1R**	**activation of CB2R**	**activation of CB1R/CB2R, with deactivation of CB1R**	**activation of CB1R/CB2R with deactivation of CB2**	**activation of CB2R and deactivation of CB1R**	**activation of CB1R and deactivation of CB2R**	**DMSO**
***NAPE-PLD***	**Highest**		**High**	**High**		**Lowest**			**High**	
***FAAH***				**Low**	**Lowest**	**Highest**		**High**	**High**	
**Primary F**	**High**		**High**				**Low**	**High**	**High**	**Lowest**
**Secondary F**		**High**		**Lowest**		**High**			**Highest**	
**Preantral F**		**High**	**Highest**				**Low**	**Low**		**Lowest**
**Antral F**		**Lowest**	**Highest**	**High**			**High**			**Lowest**
**Luteal body**					**Highest**	**High**			**High**	**Lowest**
**Microvessel**		**Highest**		**Lowest**						
**Volume**			**High**	**Low**			**Low**	**High**	**High**	
**Weight**			**High**							**Highest**
**Estradiol**			**Highest**							**Lowest**

The scoring in this table was performed visually, based on the relative height of the graphs compared to other groups; CB1R: cannabinoid receptor 1, CB2R: cannabinoid receptor 2

F: Follicle

## Conclusion

According to our data, activating or blocking CB1 and CB2 receptors may change the amount of cannabinoids in the ovary by changing the level of cannabinoid constructive or destructive genes (*NAPE-PLD* and *FAAH*). Generally, we conclude that the use of cannabinoids can negatively affect the ovaries and disrupt folliculogenesis and that the CB2 receptor is more involved in this process. Besides the positive role of CB1 receptor activation on the antrum formation stage of folliculogenesis, it negatively affects ovaries by reducing follicular growth and decreasing microvessels. It can also be stated that the observed effects of antagonist administration on the ovaries of mice are more pronounced than those of agonist administration. Our novel preliminary findings may pave the way for a better understanding of the ambiguous role of cannabinoids on the body organs, especially the female reproductive system, and provide initial insights into the interaction between these compounds at these levels. At last, it should be noted that although some similarities can be found between mice and human physiology, due to differences between animal kingdoms, clinical trials are needed to confirm the generalizability of the data between species. 

## Declaration

We confirm that no AI tools or technologies were used in the preparation, writing, analysis, or data generation of this manuscript. All parts of the study, including conceptualization, experimental design, data analysis, and manuscript writing, were performed manually by the authors.

## Confirmation Statement

All authors confirm that this research is supported by the Kerman University of Medical Science, Iran, which is primarily involved in education or research.
